# Perspectives in Sports Genomics

**DOI:** 10.3390/biomedicines10020298

**Published:** 2022-01-27

**Authors:** Valentina Ginevičienė, Algirdas Utkus, Erinija Pranckevičienė, Ekaterina A. Semenova, Elliott C. R. Hall, Ildus I. Ahmetov

**Affiliations:** 1 Institute of Biomedical Science, Faculty of Medicine, Vilnius University, 01513 Vilnius, Lithuania; algirdas.utkus@mf.vu.lt (A.U.); erinija.pranckeviciene@mf.vu.lt (E.P.); 2 Department of Systems Analysis, Faculty of Informatics, Vytautas Magnus University, 44248 Kaunas, Lithuania; 3 Department of Molecular Biology and Genetics, Federal Research and Clinical Center of Physical-Chemical Medicine of Federal Medical Biological Agency, 119435 Moscow, Russia; alecsekaterina@gmail.com; 4 Research Institute of Physical Culture and Sport, Volga Region State University of Physical Culture, Sport and Tourism, 420010 Kazan, Russia; 5 Research Institute for Sport and Exercise Sciences, Liverpool John Moores University, Liverpool L3 5AF, UK; elliotthall@live.co.uk; 6 Department of Physical Education, Plekhanov Russian University of Economics, 115093 Moscow, Russia; 7 Laboratory of Molecular Genetics, Kazan State Medical University, 420012 Kazan, Russia

**Keywords:** sports science, sports genetics, omics, bioinformatics, physical performance, athletes, injury prevention, gene doping

## Abstract

Human athletic performance is a complex phenotype influenced by environmental and genetic factors, with most exercise-related traits being polygenic in nature. The aim of this article is to outline some of the challenge faced by sports genetics as this relatively new field moves forward. This review summarizes recent advances in sports science and discusses the impact of the genome, epigenome and other omics (such as proteomics and metabolomics) on athletic performance. The article also highlights the current status of gene doping and examines the possibility of applying genetic knowledge to predict athletes’ injury risk and to prevent the rare but alarming occurrence of sudden deaths during sporting events. Future research in large cohorts of athletes has the potential to detect new genetic variants and to confirm the previously identified DNA variants believed to explain the natural predisposition of some individuals to certain athletic abilities and health benefits. It is hoped that this article will be useful to sports scientists who seek a greater understanding of how genetics influences exercise science and how genomic and other multi-omics approaches might support performance analysis, coaching, personalizing nutrition, rehabilitation and sports medicine, as well as the potential to develop new rationale for future scientific investigation.

## 1. Introduction

The ever-advancing number and standard of sporting competitions has led to an increased demand to improve the existing methods used by scientists to understand athletic capability and to drive the innovation of new recommendations for athletes to improve both health and athletic performance. Sport and exercise scientists study the adaptation of athletes to training and competition using a variety of physiological, biochemical and biomedical indicators. In recent decades, exercise physiologists have begun to elucidate the cellular and molecular responses to exercise that lead to adaptation at the whole-body level. In recent years, an increasing number of post-genomic analysis methods have been employed in the search for new markers in sports that can be used to quantify changes in the physiological and functional state of the human body [1,2]. Genetics, genomics and other multi-omics are among the fastest advancing scientific fields, prompting innovation in many disciplines including sport and exercise science. However, from both a scientific and a practical perspective, a number of questions remain unanswered. In particular, the domains of sports medicine, traumatology and the applied sports sciences may benefit from genomics data due to the prospect of personalizing training programs and nutrition, though there is also concern surrounding potentially illicit uses of genetic information for performance enhancement through means such as gene doping [1,2,3].

In the present review, we provide perspective on the current state of sports genomics and a range of potential applications for genomic data in elite sport. Here, we review the current understanding of genetics in modern sport, including several recent large-scale studies that have successfully identified and replicated the associations of genetic variants with athletic performance phenotypes. We subsequently explore recent studies that have focused on the potential of using genomic information to complement injury prevention and pre-participation screening. We will also address what impact the advancing fields of bioinformatics and omics approaches (such as transcriptomics, proteomics and metabolomics) are likely to have on the way we understand athletes’ physiology and whether information gained through these approaches could help to improve athletic performance.

## 2. Literature Research

A comprehensive search of the Medline and PubMed databases was conducted. The last query was on 1 December 2021. Findings were limited to English articles. Articles published before 1990 were excluded, unless no newer information had been published on a topic or their inclusion was necessary for understanding. Due to the wide spectrum of this topic of article, several different search terms were used in the following layout: (sports genetics) OR (sports genomics) OR (exercise genomics) AND (XXX). All of the articles identified were evaluated by their title and/or abstract and included if considered appropriate. Additionally, references of the articles cited were searched for to find further publications not detected by the literature search.

## 3. Sports Genomics: Actuality and Prospects

Interest in the genetic contribution to human sports performance can be traced back to the early 1970s, though the first major book on the topic, Genetics of Fitness and Physical Performance by Bouchard and colleagues, was not published until 1997 [4]. Another introductory level textbook Genetics Primer for Exercise Science and Health written by Roth arrived a decade later [5], with the first research in the field published in the 1990s through the work of Montgomery et al. [6], Rivera et al. [7,8] and others. Sports genomics has advanced considerably since the 1990s, largely due to technological advances and a consequent reduction in the costs of analysis. The latest collective monograph summarizing this progress, as well as the current knowledge of molecular genetic techniques and their applications to human performance and physiological characteristics, was published in 2019 [9]. 

Sports genomics is the scientific discipline focusing on the organization and function of the genome in elite athletes and aims to develop molecular methods that may be used for sports medical practices, personalized exercise training, nutrition prescription and the prevention of exercise-related injury and/or disease [9,10]. Recently, a new subdiscipline of the field known as kinesiogenomics has been introduced, combining kinesiology—the scientific study of human movement—and genomics [11].

Human physical performance traits (PPTs) and adaptations to exercise training both result from the interaction of multiple intrinsic and extrinsic factors, including those categorized as environmental, genetic, anatomical, physiological and psychological [1]. Common sequence variation in human genomic DNA contributes to the variability between individuals in a wide array of traits. In relation to sport and exercise, these include muscular fitness, exercise behavior, the capacity for cardiorespiratory, cardiovascular and metabolic adaptations to acute exercise bouts and the responsiveness to sustained training [1]. Athlete status itself is a highly heritable trait, with between 66% and 70% of the variance in athlete status explained by additive genetic factors and the remaining variance due to environmental factors that include training, nutrition and motivation, amongst others [12]. Many physiological and performance-related parameters have a substantial genetic component. Indeed, there is evidence that 60% of the variation in baseline aerobic capacity and cardiac function (30–40% in cardiac parameter), 70–90% of the variation in anaerobic power and 50–70% of the variation in muscular strength can be explained by genetic factors [1]. Furthermore, it is well established that many of the essential biological processes that underpin sports performance are genetically influenced, including skeletal muscle energy metabolism, mitochondrial biogenesis, the formation of muscle, bone and cartilage, tissue oxygenation, erythropoiesis and angiogenesis [9,10,13]. Consequently, the frequency of research to identify specific genes and their variants contributing to elite performance, which might explain differences in physiological capacity between elite athletes, is increasing.

In recent years, the potential to use genetic information to maximize athletic performance and prevent injuries has been proposed [10,14,15,16,17,18], as has the suggestion that specific dietary interventions may be individually tailored according to an athlete’s microbiome [3,19]. It has also emerged that epigenetic factors regulating gene expression without changes in DNA sequence have an important role in the response to exercise training and the predisposition to injury or disease. Epigenetic factors, such as DNA methylation, histone modifications and microRNAs, are tissue-specific regulators of gene expression that constitute a key link between the genotype and environment [20]. Indeed, it has been shown that epigenetic markers of training adaptation are retained in skeletal muscle even after periods of detraining [21,22], suggesting that epigenetic mechanisms underpin an intrinsic ability of the human body to “memorize” training adaptations. Recently, a new class of epigenetic marker known as the chromosome conformation signature has yielded promising results in medical research and may also be used to investigate whether changes in genome conformation (organization of the genome within three-dimensional nuclear space) are involved in regulating exercise responsiveness [23]. Whilst technological advances continue to assist in the discovery of new biomarkers relating to sport and exercise, the ability to maximize favorable aspects of the microbiome, genome, epigenome and other omics factors (e.g., the transcriptome, proteome and metabolome) to positively impact human PPTs relies on the combination of biomarker knowledge and appropriate environmental factors, such as effective training programs and disciplined lifestyle habits. Adherence to these well-established principles is likely to increase the efficacy and application of personalized medicine for the training and management of elite athletes to a greater extent than strategies based solely on molecular data [3,24].

### 3.1. Genetic Research in Sports

Considerable research has already been conducted regarding the roles of genetic and epigenetic factors in training adaptation and the determination of elite sports performance. Research investigating the genetic basis of PPTs was initiated by observational studies conducted in twins, families and pedigrees, evolving from genetic epidemiology research into the study of DNA sequence biomarkers [1,4]. The first achievement in human sports genetics was the determination of heritability indices of individual structural and functional traits. Twin and family studies demonstrated that many exercise-related traits, such as anaerobic power, maximal oxygen uptake, work capacity and muscle fiber type composition, are partially heritable [1,11]. Another major accomplishment has been the identification of genetic markers associated with athletic performance and the response to training [25,26,27,28,29]. The identification of such markers allowed researchers to target genetic variants that were associated with performance phenotypes in order to speculate their functional role in human performance based on each variants’ influence on specific physiological processes. For example, variants of the angiotensin-converting enzyme (*ACE*) gene, which is a known regulator of blood pressure, have been shown by multiple studies to be a determinant of endurance potential in athletes. Other examples of candidate genes that have received notable attention, and which have subsequently been associated with phenotypes underpinning sprint, power or endurance performance, include the skeletal muscle isoform of α-actinin 3 (*ACTN3*), adenosine monophosphate deaminase (*AMPD1*), muscle creatine kinase (*CKM*), insulin-like growth factor (*IGF1*) and the family of peroxisome proliferator-activated receptor (*PPAR*) genes. The associations of common genetic variants in these and other genes with a range of athletic performance phenotypes are comprehensively reviewed elsewhere [1,9,10,13,25,26,27,28,30,31,32].

At end of 2021, the total number of DNA polymorphisms associated with athlete status was 220, of which 97 markers have been found significant in at least two studies (35 endurance-related, 24 power-related and 38 strength-related) [10]. Furthermore, 29 genetic markers have been linked to soft-tissue injuries in at least two studies [10].

**Figure 1:**
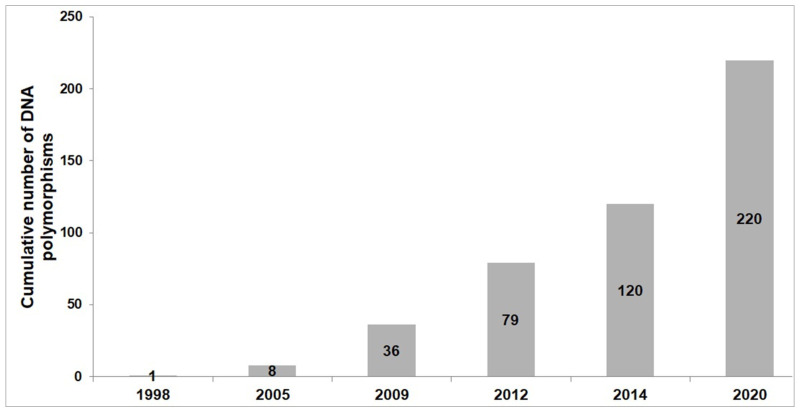
Sports-related DNA polymorphisms discovered between 1998 and 2020.

Figure 1 presents the cumulative number of sports-related DNA polymorphisms discovered from 1998 to 2020. The most promising genetic markers include *HFE* rs1799945, *MCT1* rs1049434, *MYBPC3* rs1052373, *NFIA-AS2* rs1572312, *PPARA* rs4253778, *PPARGC1A* rs8192678 and *VEGFR2* rs1870377 for endurance; *ACTN3* rs1815739, *AMPD1* rs17602729, *CPNE5* rs3213537, *CKM* rs8111989 and *NOS3* rs2070744 for power; *LRPPRC* rs10186876, *MMS22L* rs9320823, *PHACTR1* rs6905419 and *PPARG* rs1801282 for strength; and *COL1A1* rs1800012, *COL5A1* rs12722, *COL12A1* rs970547, *MMP1* rs1799750, *MMP3* rs679620 and *TIMP2* rs4789932 for soft-tissue injuries [10,17,33,34,35,36,37,38].

To date, studying the genetic profiles of elite athletes has revealed DNA sequence variations that are associated with a competitor’s predisposition to excel in strength, power, sprint or endurance sports, their intrinsic vulnerability to sports-related injuries and their individualized nutritional requirements [2]. The majority of associations with PPTs relate to common genetic variants such as single-nucleotide polymorphisms (SNPs) and indels. Though other sources of variability including copy-number variants and microRNAs have thus far received less attention, the potential role of these markers in sport and exercise merits further investigation [1,2]. The candidate gene approach, which accounts for the majority of sports genomics research to date, frequently investigates variants initially identified in animal studies using models such as gene knockout, gene knockdown and transgenic mice. These approaches have identified many candidate genes subsequently analyzed for their potential contribution to human performance phenotypes [1]. For example, mutations in the myostatin (*MSTN*) gene lead to extreme growth of skeletal muscle, indicating that myostatin is a key regulator of muscle mass [39]. Specifically, *MSTN* mutations that lead to an inactive gene product have been shown to produce double skeletal muscle mass in mice, sheep and cattle. Such knowledge may be highly valuable in helping to explain the underlying causes of certain diseases and may subsequently aid the development of new treatment methods. Indeed, increased myostatin expression might contribute to the loss of muscle mass through normal aging, suggesting that exercise modalities known to reduce myostatin expression and/or pharmacological inhibition of myostatin, might represent suitable treatments designed to prevent the age-related loss of muscle mass in non-athletic cohorts [40]. The candidate gene approach has yielded significant progress since the emergence of sports genomics. However, recent technological advances mean that more sophisticated and powerful approaches have become increasingly accessible to research institutions.

### 3.2. High-Throughput and Collaborative Approaches

The majority of sports genomics studies to date have adopted the candidate gene approach. This involves investigators selecting genes and variants they believe are related to a PPT of interest. Researchers first conduct case–control association studies to determine whether a genotype or allele is more prevalent in a cohort of athletes than a control group containing healthy non-athletes from the general population [1,9]. Typically, candidate genes are identified using known metabolic pathways or by gene expression experiments, with the subsequent case–control analysis performed on a single genetic marker [1,9,10]. However, PPTs are polygenic, meaning they are affected by numerous genes of which many are either poorly characterized or unknown [1,5,9], indicating that many candidate gene variants are yet to be discovered. This particular constraint can be overcome by analyzing whole genomes rather than individual gene variants [41]. Accordingly, genome-wide association studies (GWAS) have emerged as a high-throughput alternative that can circumvent some limitations of the candidate gene approach.

The most up-to-date GWAS platforms can screen more than one million DNA loci simultaneously in order to detect regions associated with traits of interest. One of the advantages of the GWAS approach is that it is unbiased with respect to genomic structure and previous knowledge of the trait (hypothesis-free), in contrast to candidate gene studies where a priori knowledge is used to identify candidate loci contributing to the trait of interest. In addition, some GWAS platforms enable the investigation of epigenetic modifications. To date, four GWAS have been performed in athletes [37,42,43,44].

Despite the obvious advantages of the GWAS approach, an overarching limitation of these studies is that in some countries, and in certain sporting disciplines, there is a limited number of elite athlete cohorts (cohort homogeneity). This reflects another common constraint within exercise genomics, which is that research is often conducted using small sample sizes. Accordingly, major collaborative efforts are required to increase participant numbers and sufficiently advance the impact of exercise genomics research [45]. In recognition of this need, the ATHLOME Project Consortium was established in 2015 [46], consisting of 15 participating centers worldwide that collectively study the genotype and phenotype data of elite athletes to advance current knowledge regarding the molecular basis for sports performance. The main focus of ATHLOME is to understand how athletes adapt to training, the underlying causes of exercise-related injuries and the epigenetic alterations that impact athletic performance and adaptation. Ultimately, the goal of the Consortium is to create guidelines that can inform strategies for personalized training, injury prevention and doping detection [46]. The increasing collaboration of multiple research centers also improves the ability to integrate a range of omics approaches. Indeed, current omics measurement platforms have enabled the comprehensive phenotyping of athletes’ physical performance according to variables including their level of fitness, exercise intensity and sporting discipline. As well as complementing the growing use of GWAS in sports genomics, greater accessibility to omics approaches will also increase the scope for investigating the molecular underpinnings of athletic performance.

### 3.3. Multiple Omics Approaches to Physical Performance Research

**Figure 2:**
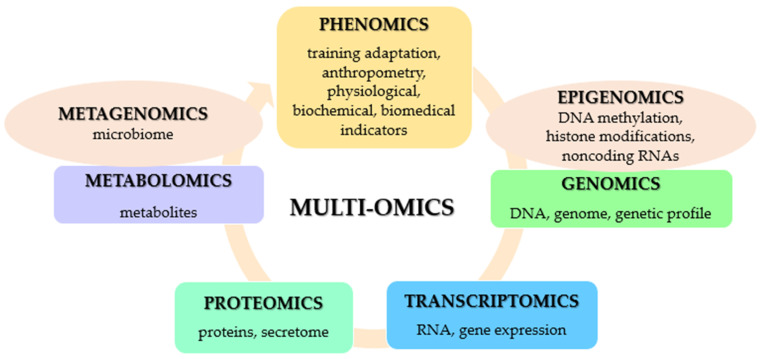
Integrating multiple omics approaches for the study of physical performance traits in athletic populations.

In the field of sport performance research, genomics focuses on identifying genetic variants associated with PPTs, response to exercise or future prognosis of injury. DNA, RNA, protein and metabolite often have complementary roles in PPTs to jointly perform a particular biological function. High-throughput technologies enable omics studies (in metabolomics, transcriptomics, proteomics, epigenomics, etc.) and provide researchers with a greater understanding of the flow of information, from the original process of PPTs biology (genetic or environmental) to the functional consequences. Multi-omics approaches integrate data obtained from different omics levels to understand their interrelation and combined impact on the biological mechanisms of physical performance. Figure 2 illustrates the method of integrating multiple omics approaches for the study of PPTs in athletic populations. Genomics is the most mature discipline of the omics fields. The latest research using transcriptomic measurements has revealed how genomic and epigenomic mechanisms affect the transcription of genes regulated by exercise, helping to identify potential biomarkers of training adaptation as well as the health benefits achieved from sports participation [21,22,23].

Epigenetics is a rapidly advancing field within molecular exercise physiology, referring to changes in gene function in the absence of changes in DNA sequence [21,22]. Epigenetic modifications (e.g., DNA methylation, histone acetylation, noncoding RNAs) regulate gene expression (by the activation or “silencing” of genes) and often occur in response to environmental and/or external stimuli, such as a specific training stimulus or diet, and induce specific changes to the transcriptional response [9,21,23]. For example, exercise has been shown to result in DNA hypomethylation in the promoter regions of key exercise-regulated metabolic genes (e.g., *PPARGC1A*, *PPARD*), accompanied by a concomitant overexpression of those genes [22]. Thus, the variability in phenotypic adaptation induced by physical activity may be explained, in part, by epigenetic factors. However, the specific epigenetic modifications elicited by exercise are not well elucidated. A prominent challenge for scientists remains to identify what type, intensity and frequency of exercise is required to maintain an epigenetic memory (e.g., at the DNA methylation level) of exercise adaptation that can be conserved for an extended period of time. Should such advances be made, it is possible that this information could be used to optimize athletic training programs or improve the process of recovery from injury [21,22,23].

Proteomic and metabolomic research methods offer a quantitative measurement of metabolic profiles associated with exercise, with some researchers attempting to identify the distinct metabolic profiles of athletes from a range of different sports. Metabolomics studies characterize exercise-related adaptations that affect an abundance of small-molecule metabolites serving a wide range of biological functions, such as biomarkers of cardiometabolic fitness in skeletal muscle [47]. “Molecular biomarkers” is a term that often includes proteins, amino acids, lipids, carbohydrates and peptides. Collectively, these metabolites form the molecular pathways underlying whole-body physiological processes such as energy homeostasis, hormonal balance, nutritional metabolism and oxidative stress mechanisms. For instance, differences in serum amino acid profiles and metabolites related to energy production and oxidative stress are evident between trained and untrained populations [10]. Intense training leads to increased levels of metabolites associated with carnitine metabolism and fatty acid metabolism, while short-term exercise induces changes in the presence of metabolites related to both the consumption and production of energy by skeletal muscle. It is also possible that biomarkers of oxidative stress, muscle damage and energy deficiency may be utilized in the future to complement strategies designed to prevent injuries [10].

The application of proteomics and metabolomics to sports research has already revealed a number of functional proteins and metabolic pathways that are altered by exercise in general or which demonstrate variable responses between different sporting disciplines. The majority of changes have been detected in cytoskeletal structure or within energy-related pathways. In addition, omics approaches have recently allowed scientists to uncover the transducers and profiles of secretomes (e.g., myokines, adipokines and hepatocines), that is, the biologically active substances secreted from cells and tissues (such as skeletal muscle, adipose and hepatic tissues) via autocrine, paracrine and endocrine systems, as an emerging subdiscipline of proteomics to study exercise-regulated signals [9,10]. However, the secretion of various myokines is dependent on the specifics of muscle activity, such as the intensity and duration of exercise, muscle fiber composition and the metabolic status of skeletal muscle, and remains poorly investigated. The study of whether and how myokines, adipokines and hepatokines interact is likely to create new approaches to addressing the training problems and health of athletes. Finally, the composition of gut microbiota has been investigated in metagenomics, with a number of potential implications for sports performance, exercise adaptation and wellness [48]. Although the interactions of gut microbiota and diet have been studied extensively, exercise microbiota studies are an emerging field of research that will soon require the integration and understanding of metagenomic data [9,10].

A key objective for integrating omics technologies into sports science is the prospective ability to prescribe personalized nutrition and training programs to athletes. Understanding the genetic, epigenetic and epigenomics underpinnings of an athlete’s nutritional requirements may provide a valuable tool to further optimize his or her sporting performance [3]. In addition, researchers have proposed that the integration of omics data may facilitate the identification of biomarkers that indicate physical fitness, fatigue, overtraining, chronic stress, inflammation or cardiovascular risk. Furthermore, the omics approach may one day be complementary to the current methods used to study an athlete’s state of physical performance and injury susceptibility and may also hold potential for the prevention and rehabilitation of sports-related injuries [16,49]. Indeed, the negative effect of injury on athletes’ availability to train and compete suggests that information pertaining to the intrinsic risk of suffering an injury may be desirable, in addition to knowledge regarding any potential contraindications to strenuous exercise.

### 3.4. Injury Prevention and Pre-Participation Screening

Injury prevention is a priority for athletes, coaches and practitioners of sports medicine for whom the diagnosis, treatment and prevention of injuries constitute a large part of a daily practice. Exercise-induced injuries to the musculoskeletal system depend on race, age, body composition, physical activity/inactivity levels and their multifactorial interaction with other extrinsic and intrinsic factors. An individual’s genetic profile is a recognized intrinsic factor predisposing individuals to a higher risk of suffering a musculoskeletal injury and/or necessitating longer recovery after intense exercise [17]. Recently, specific genetic markers have been associated with the risk of exercise-related injuries [10,15,50]. For example, candidate gene association studies and GWAS have identified variants of collagen-encoding genes (*COL1A1*, *COL3A1*, *COL5A1* and *COL12A1*) and matrix metalloproteinase-encoding genes (*MMP12* and *MMP3*) that are associated with injuries such as Achilles tendon pathology and anterior cruciate ligament rupture [10,15,16,49]. It follows that information relating to an individuals’ genotype for these and other variants could one day be useful in the design of injury prevention strategies through enhanced training prescription and/or the optimization of post-injury rehabilitation and therapeutics [10,14,15,16,17,18].

In addition to the problem caused by sports injuries, there are a range of genetic conditions that may unknowingly increase the risk to an individual’s health when participating in exercise. The most severe examples include those that elevate the risk of sudden cardiac death (SCD). In this context, genetic data could potentially be applied during pre-participation screening for the identification of at-risk individuals and the prevention of SCD in sport. SCD refers to the unexpected natural death of an individual that is caused by cardiac arrest within a brief time interval (generally ≤1 h from first symptoms) under conditions that would not normally be considered fatal, and it is the leading medical cause of death in young athletes during sport or exercise activity [51,52,53]. Hypertrophic cardiomyopathy is the most common cause of SCD in athletes, accounting for 35% of confirmed cases in the United States [51], and most cardiac abnormalities that predispose an athlete to SCD are inherited, quiescent disorders [51,52]. In this context, genes associated with cardiomyopathies have logically attracted the interest of sports medicine physicians [53]. Mutations in more than 30 genes including *TTN*, *ABCC9*, *DMD*, *MYPN* and *SCN5A*, amongst others, have been implicated in the causes of familial cardiomyopathy. Athletes in soccer, basketball, cross-country and long-distance running are most vulnerable to suffering SCD [51]. Whilst SCD in an athlete is rare, it is a tragic and devastating event for athletes, teams, leagues, families and wider communities [51,52], which underlines the potential importance of an individual athlete’s genetic profile in assessing their risk of SCD.

### 3.5. The Use of Bioinformatics and Data Analysis Methods in Sport Genomics

High-quality experimental data and accurate analysis continue to advance the understanding of the genetic and molecular basis of a range of human PPTs including sprint, strength and endurance performance. Here, we briefly describe a number of databases, as well as computational and statistical methods, that can be used when researching genetic markers related to PPTs.

Currently, several approaches are routinely employed to identify genetic variations affecting sport-related traits, including SNP array-based GWAS and next-generation sequencing (NGS) [54]. GWAS uncover genome-wide markers associated with a trait of interest using very large cohorts including “cases” (e.g., elite athletes) and controls (e.g., non-athletes). The NGS approach is typically used in functional analysis studies to measure RNA or protein expression before and after an imposed stimulus, such as exercise or a nutritional or pharmacological intervention [54]. A catalogue of GWAS is maintained by the European Molecular Biology Laboratory and Bioinformatics Institute (EMBL-EBI, https://www.ebi.ac.uk/gwas/, accessed on 20 November 2021) to document genome-wide associations for PPTs and other traits of interest [55]. In addition, functional gene expression data from humans and other organisms obtained through the use of NGS is publicly available from the National Center for Biotechnology Information (NCBI) in concert with the Gene Expression Omnibus (GEO) and Sequence Read Archive (SRA) repositories [56]. Among these databases are details of functional studies related to PPTs, physical fitness and exercise responsiveness in human and model organisms. Data mining of these publicly accessible resources may provide important experimental evidence for the selection of new candidate genes to be investigated for an association with PPTs.

There are two broad categories of computational and statistical methods in existence that are used to infer associations between genetic markers and PPTs: (i) methods for individual marker–trait association inference and (ii) methods utilizing polygenic scores to infer predispositions to enhanced physical fitness or sport-specific phenotypes. The first class of methods rely on simple statistical techniques that compare genotype and/or allele frequency distribution between cases and controls. For categorical traits, such as endurance versus sprint athlete status or power athlete status versus non-athlete control status, chi-square, Fisher’s exact or logistic regression tests are typically employed [13,31,32,57]. For continuous traits, such as VO_2_ max, strength and/or specific anthropometric indicators of power output, associations with genetic markers are inferred using regression analysis or one-way analysis of variance (ANOVA) [7,58,59,60,61]. In many cases, these simpler methods are preferred to the more complex polygenic scoring methods because of the limitations associated with analyzing relatively small samples of elite athletes.

In 2008, Williams and Folland [62] presented a new approach to investigating additive trait outcomes: a total genotype score (TGS). This approach has also been referred to as a polygenic score [63,64] or genetic predisposition score [65]. The TGS method sums the effects of multiple genotypes that favor a certain trait to provide a quantitative measure of an individual’s predisposition to the investigated trait that is based on their genetic profile. Based on the general assumption that for a given genetic marker “j” the “AA” genotype is the trait-predisposing genotype, the “Aa” genotype is neutral, and the “aa” genotype is the unfavorable or trait-lacking genotype, the TGS method assigns scores to each genotype as follows: S^j^_AA_ = 2, S^j^_Aa_ = 1 and S^j^_aa_ = 0. Once scored, the sum of genotypes across an infinite number of variants can be calculated for each individual participant, meaning that possessing a greater number of trait-predisposing alleles from multiple variants will result in a higher TGS, indicating a greater predisposition to the trait of interest. For the total number of markers (N) included, the total genotype score is computed as: TGS = 100 × (Σ_j=1…N_S^j^/2 × N). Subsequently, the discriminatory power of a TGS can be investigated in cohorts of athletes and/or controls in relation to a categorical or continuous trait. Where a statistically significant association with a trait can be established using individual markers in the first instance, a TGS can then be computed from that subset of markers, demonstrating statistically significant associations for each individual in order to quantify and score their polygenic predisposition to the trait of interest. Since the TGS concept was introduced, the approach has been adopted, and at times adapted, by several studies [64,66]. For example, a modified TGS used when studying Israeli athletes provided evidence that power/sprint athletes can be differentiated from endurance athletes using polygenic scores [67]. Whilst the TGS approach is limited by including a relatively small number of gene variants, the approach provides greater context than the investigation of single genes or variants in isolation. However, it is also important to note that the likelihood of an elite athlete possessing the optimal genotype for every investigated polymorphism deceases exponentially as the number of included variants is increased [62], suggesting that the chances of any athlete in the world population having the optimal genotype combination for every performance-associated variant is almost impossible. It should be noted that the polygenic profiles of athletes may be affected by a phenomenon known as epistasis (or SNP–SNP interactions). These interactions occur when a combination of two or more SNPs affects a phenotype to a greater extent, or in a different way, than the effect seen with an individual gene. For example, an epistatic effect of two SNPs (*SNAP25* rs362584 and *ACTN3* rs1815739) has been described in gymnasts [68].

An additional problem arising from the relatively small samples of elite athlete populations is the limited ability to obtain strong evidence of genotype–phenotype associations. In such cases, published data of specific gene markers from multiple populations can be pooled in a meta-analysis to provide a stronger evidence based for genotype–phenotype associations [38,69]. In a meta-analysis, the odds ratios of marker–trait associations reported by multiple studies are pooled together to compute the average odds ratio and its 95% confidence interval. Use of the meta-analysis approach also helps to resolve the issue of heterogenous associations across different populations by pooling multiple studies to determine whether a specific variant should be considered favorable or unfavorable in relation to a trait of interest [70]. When access to more powerful high-throughput methods is limited, the meta-analysis approach can offer more robust and contextual results than single-variant candidate gene studies.

### 3.6. New Genetic Technologies and Gene Doping in Sports

Advancing knowledge surrounding the genetic variants that predispose individuals to success in specific sporting domains also creates the risk that such information could be used for illegal doping. New technologies for the transfer of genetic material (DNA/RNA), known as a gene therapy, are actively being developed to treat severe disorders such as anemia, peripheral vascular diseases and muscular dystrophy [24,71]. Despite the fact that the original intended use of gene therapy is for ethical purposes, and that this technique may have potentially broad applications in sport traumatology for healing traumas using specific growth factors (such as *bFGF* or *NGF*), this procedure could also be abused by athletic populations. However, understanding how gene therapy may be utilized for elicit purposes also allows experts to design new strategies to prevent doping in sport. For example, researchers have discovered that copy number variation in the *UGT2B17* gene influences the efficacy of anti-doping methods and that tests designed to detect testosterone abuse may be less sensitive in athletes of some geographical ancestries where certain genotypes are more frequent [24,71]. In such instances, additional measures including individual, genotype-based testosterone thresholds can be used to improve the ability to detect doping violation.

Gene doping refers to the non-therapeutic use of gene therapy by healthy athletes to improve physical performance in sporting competition. Gene doping could have dangerous and even fatal outcomes, as the knowledge of gene therapy is still in its infancy [72]. Through genetic modification, exogenous DNA (or RNA) sequences are inserted into specific tissues to alter gene activity and protein expression. Current knowledge suggests that the exogenous genes that are most likely to be utilized for gene doping practices include erythropoietin (*EPO*), vascular endothelial growth factor (*VEGFA* and *VEGFD*), insulin-like growth factor 1 (*IGF-1*), growth hormone (*GH*), phosphoenolpyruvate carboxykinase 1 (*PCK1*), myostatin (*MSTN*) and *MSTN* antagonists such as follistatin (*FST*) [71]. These genes have been specifically identified based on a growing body of evidence that suggests *MSTN, IGF, GH* and *rhGH* (recombinant human *GH*) are the major determinants of successful performance in sprint/power sports, with *EPO* (and *rEPO*), *VEGFA*, *HIF-1*, *PPARD* and *PCK1* associated with elite performance in endurance sports [71]. In 2003 the World Anti-Doping Agency (WADA) added gene doping to the list of prohibited practices, before deliberating the risks of gene doping and announcing funding for a research program to identify methods for doping detection [73,74]. According to the 2013 and 2021 list of prohibited practices, the WADA defines gene doping as “the transfer of polymers of nucleic acids or nucleic acid analogues or the use of normal or genetically modified cells” [75,76]. Whilst potentially desirable to a small number of unscrupulous athletes, the non-therapeutic use of gene transfer technologies solely for performance enhancement poses many risks to an athlete’s health. The most notable risks of using gene therapy for the purposes of gene doping include cancer, negative autoimmune response to an extraneous protein and an adverse reaction to the carrier virus [24,71]. The most relevant candidate genes for gene doping and their potential risk to the health of athletic populations are summarized in Table 1.

The current strategy to screen for gene doping involves the direct detection of cDNA sequences, and anti-doping laboratories must permanently incorporate new detection techniques in order to maintain a harmonized approach to detecting doping substances and methods on the worldwide scale [71].

At present, the clustered regularly interspaced short palindromic repeats (CRISPR/Cas) system is one of the most frequently used tools for genome editing. It enables the specific modification of any desired DNA sequence and surpasses all existing alternatives for gene editing [77]. However, these advantages also potentially facilitate the illegal use of the CRISPR/Cas system in sports. This abuse is classified as gene doping, which is banned in sports according to the Prohibited List of the WADA. Interestingly, while being a means of illicit gene editing, CRISPR/Cas has also been used as a tool to detect gene transfer-based doping attempts [77,78].

In 2021, WADA released guidelines concerning gene doping detection methods (e.g., using polymerase chain reaction) [79] and several studies elaborating on currently available models (e.g., mouse model) and analytical approaches for detecting transgenes for sports drug testing purposes [80,81]. The rapidly evolving options in gene editing will necessitate different analytical approaches to contain the impending issue of gene doping [78].

## 4. Conclusions and Perspective

Exercise and sport-specific training induce the stimulation of many biological networks contributing to the complex yet coordinated response of the human body to perturbations in homeostasis and mechanical demand. Developments of omics technologies have created new possibilities in the study of the molecular mechanisms underlying athletic performance. Technological advances in genome-wide association studies and the profiling of gene expression have each contributed to our understanding of the genes, pathways and biological networks involved in the response to exercise, as well as the presence of variability in training responses among individuals. Sports genetics and genomics may one day help individuals to achieve superior outcomes in their general health and wellness. In athletic settings, genetic information has the potential to identify individuals with a natural predisposition to achieving success in specific athletic activities and may also be useful for athletes seeking to understand and make improvements in specific performance attributes where their genetic predisposition may be less favorable. In addition, there is scope for genetic information to assist in strategies aimed at reducing the risks of injury and to augment the process of rehabilitation once an athlete suffers an injury. Further collaborative research is needed to confirm the potential influence of genetic variants in athletes from different sporting backgrounds to identify more informative markers of athlete health and performance. The use of genetic testing in sports presents a number of possibilities for athletes, coaches and medical personnel to understand athletes’ susceptibility for specific pathological conditions including sports injuries, sudden death and cardiomyopathies, among others. With improved study designs and the increasing availability of advanced analytical tools and statistical approaches, the interaction of genetic variability with sport and exercise can be better understood and potentially utilized to improve human health, wellbeing and performance. Over the next decade, the application of genomics in sport is likely to progressively improve through the increasing availability of complete omics databases and high-throughput screening approaches for investigating human performance.

## Figures and Tables

**Table 1:** Genes with the potential to enhance athletic performance and their potential adverse health effects [24,71,72], and information about physiological functions from The Human Gene Database (https://www.genecards.org/, accessed on 22 January 2022).

Gene(s)	Molecular Mechanisms and Physiological Functions	Athletic Performance Enhancement (Phenotype)	Adverse Health Effects
Erythropoietin (*EPO*) and EPO receptor (*EPOR*) genes	Stimulates erythropoiesis, increases hemoglobin and hematocrit levels, enhances blood oxygenation and oxygen delivery to tissues	Endurance	Hyperviscosity, restricted blood flow, severe immune response, stroke, thrombosis, hypertension, myocardial infarction, heart failure
Peroxisome Proliferator Activated Receptor Gamma Coactivator 1-Alpha (*PPARGC1A*) and -Beta (*PPARGC1B*) genes	Stimulates the activity of transcription factors and nuclear receptors; regulates the genes involved in energy metabolism and mitochondrial biogenesis; regulates muscle fiber type determination	Strength and endurance, greater resistance to fatigue	Metabolic disorders, mitochondrial diseases
Peroxisome Proliferator Activated Receptor Delta (*PPARD*) gene	Regulates energy homeostasis, muscle fiber type composition and fatty acid catabolism (with a broad role in fat metabolism)	Sprint and endurance	Metabolic disorders, colorectal cancer, overexpression of sex hormones
Vascular Endothelial Growth Factor A (*VEGFA*) gene	Induces proliferation and migration of vascular endothelial cells, and is essential for angiogenesis	Endurance	Abnormal blood vessel formation, cancer, immune system disorders
Hypoxia Inducible Factor 1 Subunit Alpha (*HIF1A*) gene	Regulates metabolic adaptation to hypoxia, energy metabolism, angiogenesis and apoptosis	Endurance	Hyperviscosity, hypertension, heart failure, neoplastic and ischemic disease
Insulin-Like Growth Factor 1 (*IGF1*) and Growth Hormone (*GH*) genes	Involved in mediating growth and development of bones and tissue mass, muscle hypertrophy and hyperplasia, homeostasis of carbohydrates, proteins and lipids; regulates muscle regeneration and increased release of glucose from liver	Strength, power, increase in muscle mass, positive effect on muscle regeneration	Hypertension, neoplastic disease, cardiomyopathy, insulin resistance and diabetes, overgrowth of the cartilage of the nose and jaw, abnormal vision, peripheral oedema, carpal tunnel syndrome, nausea and vomiting, headache, musculoskeletal pain
Myostatin (*MSTN* or Growth Differentiation Factor 8, *GDF8*) gene	Negatively regulates skeletal muscle cell proliferation and differentiation	Strength, increase in muscle mass	Musculoskeletal disorders
Angiotensin-Converting Enzyme (*ACE*) gene	Involved in blood pressure regulation and electrolyte balance	Endurance and/or sprint	Kidney and cardiovascular disease, angioedema
Alpha-Actinin 3 (*ACTN3*, Skeletal Muscle Isoform) gene	Expressed in skeletal muscle (fast-twitch myofibers) and functions as a structural component of sarcomeric Z line	Sprint and/or endurance	Data currently unavailable

## Data Availability

Not applicable.
